# Natural Alkaloids Against Liver Injury: Mechanistic Insights and Multi-Target Therapeutic Potential

**DOI:** 10.3390/biom16060815

**Published:** 2026-05-31

**Authors:** Yating Xiao, Zhirui Tian, Jie Cui, Xia Yang, Dong Wang, Yangyang Cai, Qian Wu, Yaxin Jin, Hua Chen, Kai Ji

**Affiliations:** Key Laboratory of Protection, Development and Utilization of Medicinal Resources in Liupanshan Area, Ministry of Education, School of Pharmacy, Ningxia Medical University, No. 1160 Shengli Street, Yinchuan 750004, China; xiaoyating2583@nxmu.edu.cn (Y.X.); wangming@nxmu.edu.cn (Z.T.); cuijie@nxmu.edu.cn (J.C.); yangxia@nxmu.edu.cn (X.Y.); wangdong@nyfy.com.cn (D.W.); yangycai@nxmu.edu.cn (Y.C.); wuq@nxmu.edu.cn (Q.W.); asdfghjkl@nxmu.edu.cn (Y.J.)

**Keywords:** liver injury, natural alkaloids, hepatoprotection, signalling pathways, drug development

## Abstract

Liver injury is a complex pathological condition with increasing incidence due to diverse etiologies, including drug-induced liver injury, metabolic dysfunction-associated steatotic liver disease, alcoholic liver disease, viral hepatitis, and autoimmune disorders. Its progression is characterized by persistent hepatic damage and gradual loss of liver function, which may ultimately lead to fibrosis, cirrhosis, and liver failure. However, currently available hepatoprotective drugs still present several limitations, such as insufficient target specificity, limited therapeutic efficacy, and potential adverse effects, highlighting the need for safer and more effective alternatives. Based on a comprehensive search of databases including PubMed, Web of Science, China National Knowledge Infrastructure and Google Scholar, this review summarizes the pathogenic mechanisms of major liver injury types and the therapeutic potential of natural alkaloids. As key secondary metabolites of medicinal plants, alkaloids exhibit structural diversity, potent bioactivities, and favorable safety profiles. Increasing evidence suggests that natural alkaloids exert hepatoprotective effects through multi-target and multi-pathway mechanisms, including the regulation of oxidative stress, inflammation, lipid metabolism, and cell death. These findings highlight their promising potential for the prevention and treatment of liver injury and provide a theoretical basis for the development of novel hepatoprotective agents.

## 1. Introduction

Liver disease claims the lives of about 2 million people each year, or 4% of all fatalities worldwide [1]. The most common clinical types of hepatic damage are drug-induced liver injury (DILI), alcoholic liver disease (ALD), metabolic dysfunction-associated steatotic liver disease (MASLD), viral hepatitis, and autoimmune liver diseases (AILD) [2]. The incidence of liver injury has shown an annual upward trend due to the widespread use of antibiotics, antineoplastic agents, and traditional Chinese herbal medicines, as well as the increasing prevalence of metabolic syndrome and obesity [3]. In mainland China, the annual incidence of DILI was estimated to be 23.80 per 100,000 people, which is higher than that reported from Western countries [4].

Currently, N-acetylcysteine, ursodeoxycholic acid, and silymarin are commonly used agents in the clinical management of liver diseases [5,6], employed for specific liver-related indications or as adjunctive agents. While these medications demonstrate efficacy in targeted indications or symptomatic relief, their clinical utility is largely restricted to particular etiologies, and most serve only as adjunctive rather than disease-modifying therapies. Notably, conventional treatment approaches may be limited in fully targeting the complex, multi-layered pathogenic networks of oxidative stress, inflammation, and metabolic dysregulation common to various forms of liver injury [7]. This gap highlights an urgent need for novel therapeutic strategies capable of targeting these shared core mechanisms to provide broad-spectrum hepatoprotection.

This clinical imperative has directed research focus towards natural alkaloids. Characterized by their specific nitrogenous heterocycles, alkaloids possess diverse pharmacological activities that are distinct from conventional single-target therapeutics. Uniquely, alkaloids function via a holistic ‘multi-target’ strategy that aligns well with the multifactorial nature of liver injury. They can simultaneously modulate key signaling networks (e.g., Nrf2/ARE, NF-κB, and PI3K/AKT) that are universally activated across various liver pathologies [8]. For instance, alkaloids like oxymatrine and berberine have demonstrated efficacy in models of DILI, MASLD, and hepatitis by regulating shared pathological nodes such as inflammasome activation and mitochondrial dysfunction.

## 2. Literature Search Strategy and Selection Criteria

A literature search was conducted in PubMed, Web of Science, China National Knowledge Infrastructure, and Google Scholar for relevant publications available up to 28 February 2026. Studies related to liver injury, natural alkaloids, hepatoprotection, molecular mechanisms, and therapeutic strategies were considered for inclusion. Eligible original research articles included in vitro experiments and in vivo studies using animal models. Review articles were used primarily to provide background information and to identify additional relevant primary studies. Articles published in English or Chinese were considered.

Studies were included if they investigated the protective effects, pharmacological activities, or molecular mechanisms of natural alkaloids in liver injury-related conditions, including drug-induced liver injury, metabolic dysfunction-associated steatotic liver disease, non-alcoholic fatty liver disease, alcoholic liver disease, autoimmune liver disease, and viral hepatitis. Studies were excluded if they mainly focused on alkaloid-induced hepatotoxicity, liver fibrosis, or hepatocellular carcinoma without directly addressing liver injury or hepatoprotective mechanisms. Conference abstracts without sufficient original data, articles without full-text availability, duplicate publications, and studies with insufficient methodological details were also excluded.

The selected literature was screened according to its relevance to the scope of this review. Data extraction focused on study design, alkaloid source and classification, liver injury model, experimental system, intervention characteristics, major hepatoprotective effects, underlying molecular mechanisms, and potential therapeutic implications.

## 3. Classification and Mechanisms of Liver Injury

Clinically, liver injury is primarily categorized based on disease course and etiology. According to disease duration, it is classified as acute or chronic liver injury, with the distinction primarily based on the duration of hepatic inflammation: cases lasting less than six months are defined as ‘acute’, while those exceeding six months are termed ‘chronic’ [9]. Common etiological categories include viral hepatitis, ALD, MASLD, DILI, and AILD. Liver injury caused by different etiologies exhibits significant heterogeneity in clinical severity. The pathological progression may range from mild, self-limiting liver enzyme abnormalities to fibrosis, cirrhosis, and ultimately liver failure, with some end-stage patients eventually requiring liver transplantation.

### 3.1. Mechanism of DILI

DILI refers to liver toxicity induced by a drug or its metabolite. Nonsteroidal anti-inflammatory drugs, anti-tuberculosis drugs, antiepileptic drugs, traditional Chinese medicines, and other drugs can cause varying degrees of DILI [10]. It has now become one of the leading causes of acute liver injury, liver failure, and liver transplantation worldwide. Its pathogenesis primarily involves the direct or indirect toxic effects of drugs or their metabolites on hepatocytes [11]. Clinically, numerous commonly used medications may induce drug-induced liver injury. Examples include acetaminophen (APAP), isoniazid, pyrogallol, rifampicin, diclofenac, and atorvastatin [12]. With the continuous rise in tumor incidence in China, DILI during anticancer therapy has become a critical issue affecting patients’ treatment course and prognosis, with severe cases even leading to acute liver failure or death [13,14]. Common antitumor drugs that can induce liver injury include: oxaliplatin, methotrexate, cyclophosphamide, crizotinib, pembrolizumab, nivolumab, tamoxifen, among others [15].

APAP is one of the most common causes of DILI, accounting for 15% to 57% of acute liver failure cases in Western countries [16]. Currently, research on the hepatotoxicity of APAP is relatively systematic and comprehensive, serving as a classical model for elucidating the pathogenesis of DILI. At therapeutic doses, APAP is primarily metabolized via hepatic glucuronidation and sulfation pathways into non-toxic products for excretion, thus not causing liver injury [17,18]. However, excessive intake of APAP leads to its increased metabolism via the cytochrome P4502E1 (CYP2E1), generating large quantities of the highly reactive toxic intermediate, N-acetyl-p-benzoquinone imine (NAPQI) [17]. The excess NAPQI rapidly depletes the intracellular glutathione (GSH) reserves in hepatocytes. Unconjugated NAPQI covalently binds to hepatic proteins (particularly mitochondrial proteins) to form adducts, directly impairing protein function [17,19] and inhibiting the electron transport chain (ETC). This results in a marked increase in reactive oxygen species (ROS) and peroxynitrite (ONOO^−^) levels, triggering severe mitochondrial oxidative stress [20].

**Figure 1:**
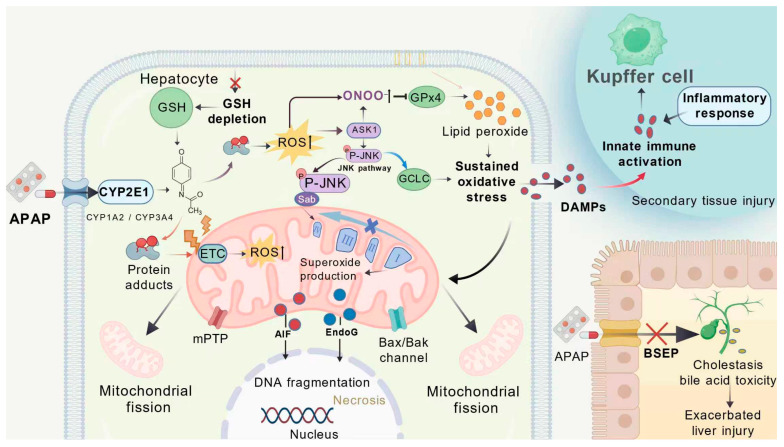
Mechanism of APAP-induced hepatotoxicity. Excessive APAP is metabolized by CYP2E1 (as well as CYP1A2 and CYP3A4) to generate reactive metabolites (e.g., NAPQI), leading to glutathione (GSH) depletion, protein adduct formation, and mitochondrial oxidative stress. Subsequent activation of the ASK1/JNK signaling pathway contributes to GPX4 dysfunction, promotes lipid peroxidation, mitochondrial dysfunction, and mPTP opening, ultimately resulting in DNA fragmentation, hepatocyte necrosis, and DAMP-mediated inflammatory responses. In addition, APAP may induce cholestasis via BSEP inhibition and bile acid toxicity. ×: Indicate inhibition. Abbreviations: AIF, Apoptosis-inducing factor; APAP, Acetaminophen; ASK1, Apoptosis signal-regulating kinase 1; Bax/Bak, Bcl-2-associated X protein/Bcl-2 antagonist/killer 1; BSEP, Bile salt export pump; CYP1A2, Cytochrome P450 1A2; CYP2E1, Cytochrome P450 2E1; CYP3A4, Cytochrome P450 3A4; DAMPs, Damage-associated molecular patterns; EndoG, Endonuclease G; ETC, Electron transport chain; GCLC, Glutamate-cysteine ligase catalytic subunit; GPx4, Glutathione peroxidase 4; GSH, Glutathione; JNK, C-Jun N-terminal kinase; mPTP, Mitochondrial permeability transition pore; ONOO^−^, Peroxynitrite; ROS, Reactive oxygen species. Created with BioGDP.com [26].

Persistent oxidative stress further activates upstream kinases (such as ASK1), leading to the phosphorylation and sustained activation of c-Jun N-terminal kinase (JNK) [21]. The activated JNK translocates to the mitochondria and binds to the mitochondrial outer membrane protein Sab, further inhibiting ETC function and resulting in massive generation of superoxide (·O_2_^−^). The superoxide reacts with nitric oxide (NO) to produce large amounts of ONOO^−^. ONOO^−^ directly oxidizes and inhibits the activity of glutathione peroxidase 4, catalyzes protein tyrosine nitration, and exacerbates mitochondrial damage. Concurrently, the reduction in glutathione peroxidase 4 activity leads to the accumulation of lipid peroxides, thereby triggering ferroptosis [22]. Furthermore, activated JNK can also degrade the rate-limiting enzyme for glutamate-cysteine ligase catalytic subunit, hindering the recovery of antioxidant reserves and perpetuating oxidative stress. Sustained oxidative/nitrative stress ultimately results in the loss of mitochondrial membrane potential, inducing mitochondrial fission and the opening of the mitochondrial permeability transition pore (mPTP) as well as the formation of Bax/Bak channels [23]. This leads to the release of pro-apoptotic factors such as apoptosis-inducing factor and endonuclease G from the mitochondria into the nucleus, causing extensive DNA fragmentation and ultimately leading to programmed cell death (PCD) [23]. The necrotic hepatocytes release damage-associated molecular patterns, which activate the innate immune system and trigger an inflammatory response, potentially causing secondary injury [24]. Additionally, APAP and its metabolites can disrupt bile duct structures, inhibit the function of the bile salt export pump, leading to cholestasis and bile acid accumulation toxicity, further aggravating liver injury [25] (Figure 1).

Beyond APAP, other common drugs induce DILI through distinct mechanisms. For instance, isoniazid undergoes metabolism via N-acetyltransferase 2 to produce toxic intermediate metabolites, leading to accumulation of protoporphyrin IX in the liver and subsequent hepatic injury [27]. Diclofenac exerts its effects through its metabolites, which directly induce cytotoxicity, trigger immune responses as haptenic antigens, cause mitochondrial dysfunction, and activate both innate and adaptive immunity in the liver. This ultimately leads to T-cell and B-cell-mediated immune-mediated liver cell injury [13,28]. Additionally, rifampicin primarily causes liver damage through mechanisms including cholestasis, endoplasmic reticulum (ER) stress, and hepatic lipid accumulation [29]. Statins exhibit hepatotoxicity and cholestatic toxicity [13]. Atorvastatin has been the most frequently implicated statin in all of the series of statin-induced hepatotoxicity [30].

Antitumor drugs are categorized into conventional chemotherapeutic agents, molecular targeted drugs, immune checkpoint inhibitors, endocrine therapy drugs, among others. The mechanisms underlying liver injury induced by different chemotherapeutic agents exhibit significant heterogeneity. Conventional chemotherapeutic agents such as oxaliplatin primarily induce hepatic sinusoidal obstruction syndrome through direct or indirect damage to hepatic sinusoidal endothelial cells, subsequently leading to sinusoidal blood flow impairment and portal hypertension. Their toxicity also involves multiple pathways including oxidative stress, lipid metabolism disorder, and intestinal flora dysbiosis [16,31]. Methotrexate primarily exerts an additive effect on pre-existing lipid metabolism disorders, indirectly exacerbating the progression of liver injury [32]. The hepatotoxicity of cyclophosphamide stems from the direct toxic effects of its active metabolites on hepatocytes, coupled with synergistic involvement of mechanisms such as oxidative stress and mitochondrial dysfunction [33]. Molecular targeted drugs such as crizotinib can induce hepatotoxicity through DNA damage, ROS generation and the mitochondrial apoptosis pathway [34].

Immune checkpoint inhibitors, such as pembrolizumab and nivolumab, primarily induce immune-mediated hepatitis resembling autoimmune hepatitis by excessively activating the immune system, which triggers immune cells to attack hepatocytes. In a minority of cases, they may also cause cholangitis [35]. Furthermore, pembrolizumab can directly damage hepatic sinusoidal endothelial cells, disrupt microvascular architecture, promote thrombus formation, and cause portal venous inflow obstruction and hypertension, ultimately leading to hepatic sinusoidal obstruction syndrome [35]. Tamoxifen primarily induces liver injury by disrupting lipid metabolism and bile acid homeostasis, including interference with the gut-liver axis and the farnesoid X receptor signaling pathway [32,36] (Table 1).

### 3.2. Mechanism of MASLD

Given that the original designation non-alcoholic fatty liver disease (NAFLD) failed to highlight the core pathogenic mechanism of metabolic dysfunction, and that the clinical definition of ‘non-alcoholic’ was ambiguous (e.g., failing to encompass cases where minimal alcohol consumption was present but metabolic disorder was the primary driver), a 2023 multi-society statement led by the American Association for the Study of Liver Diseases formally renamed NAFLD as MASLD [37]. MASLD is a chronic progressive liver disorder occurring in genetically susceptible individuals due to nutritional excess and insulin resistance. It is closely associated with metabolic syndrome, including diabetes, hypertension, obesity, and hyperlipidaemia [38,39,40]. Current epidemiological studies indicate that MASLD affects a substantial proportion of the global adult population, and its burden is expected to continue increasing in parallel with the rising prevalence of obesity and metabolic syndrome [41]. Notably, this proportion may further increase to approximately 56% in China, the United States, and most European countries. MASLD has become the most prevalent chronic liver disease globally.

**Figure 2:**
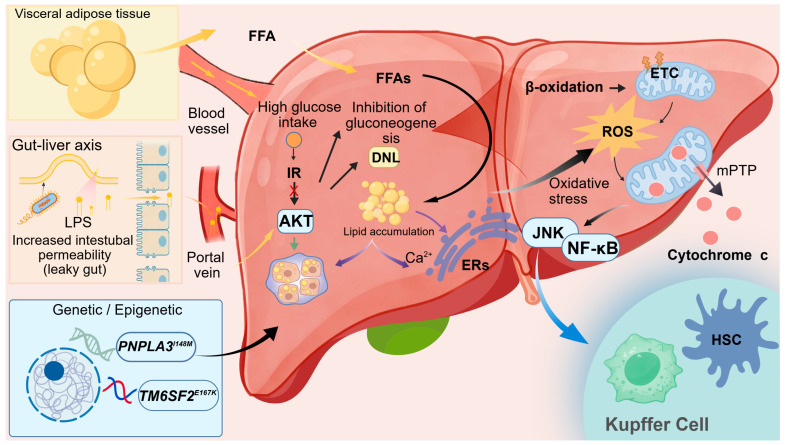
Mechanisms of MASLD. Excess visceral fat storage and high glucose intake promote insulin resistance, hepatic lipid accumulation, and de novo lipogenesis, establishing the metabolic basis of MASLD. Lipotoxicity-induced mitochondrial dysfunction and endoplasmic reticulum stress increase ROS production, trigger mPTP opening, and activate stress signaling pathways such as JNK and NF-κB, thereby promoting hepatocyte injury, Kupffer cell activation, and hepatic fibrogenesis. Gut-derived LPS, genetic/epigenetic factors, and other modulators further exacerbate disease progression. ×: Indicate inhibition. Abbreviations: AKT, Protein kinase B; DNL, De novo lipogenesis; ERs, Endoplasmic reticulum stress; ETC, Electron transport chain; FFA, Free fatty acids; HSC, Hepatic stellate cell; IR, Insulin resistance; JNK, C-Jun N-terminal kinase, LPS, Lipopolysaccharide; NF-κB, Nuclear factor-κB; *PNPLA3^I148M^*, Patatin-like phospholipase domain-containing protein 3 isoleucine-to-methionine at position 148; ROS, Reactive oxygen species; *TM6SF2^E167K^*, Transmembrane 6 superfamily member 2 glutamate-to-lysine at position 167; mPTP, Mitochondrial permeability transition pore. Created with BioGDP.com [26].

The pathogenesis of MASLD is complex, with core mechanisms including dysfunction of lipid metabolism, lipotoxicity stress, and genetics and epigenetic factors regulation [42]. Metabolic dysfunction serves as the pivotal initiating factor for MASLD. Upon saturation of visceral fat storage, enhanced lipolysis leads to a surge of free fatty acids into the liver, causing hepatic fatty acid accumulation. Concurrently, excessive glucose intake induces insulin resistance, selectively disrupting the protein kinase B (AKT) signalling pathway. This disruption simultaneously diminishes the inhibition of gluconeogenesis and excessive de novo lipogenesis, thereby further exacerbating hepatic fat accumulation, hyperglycaemia, and hyperinsulinaemia. These processes collectively constitute the metabolic basis of MASLD [39,43,44]. Lipotoxicity induced by excess lipids directly causes cellular damage and metabolic dysfunction. β-oxidation of free fatty acids leads to electron leakage in the mitochondrial ETC, generating excessive ROS and inducing oxidative stress; simultaneously, FFAs can induce ER stress, disrupting Ca^2+^ homeostasis and further exacerbating mitochondrial ROS production [45]. ROS not only causes direct cellular damage but also synergizes with ER stress to promote the opening of the mitochondrial permeability transition pore (mPTP) [42], releasing cytochrome c and inducing apoptosis. Furthermore, ROS activates stress pathways including JNK and NF-κB, driving activation of Kupffer cells and hepatic stellate cells (HSCs), thereby accelerating hepatic inflammation and fibrosis [46]. Notably, ER stress and hepatic lipid accumulation form a vicious cycle that further aggravates insulin resistance and promotes lipogenesis. Mitochondrial dysfunction, inflammatory factors, and exogenous factors further amplify this process, collectively driving disease progression [47] (Figure 2).

Moreover, intestinal microbiota increases intestinal barrier permeability, facilitating lipopolysaccharides (LPS) entry into the portal vein circulation via the gut-liver axis. This directly induces hepatocyte injury and inflammation, thereby accelerating MASLD progression [47,48]. Concurrently, genetic variants in genes such as *PNPLA3^I148M^* and *TM6SF2^E167K^* significantly elevate MASLD risk by impairing lipid metabolism and export efficiency [49]. At the epigenetic level, mechanisms including DNA methylation and m^6^A modification can regulate the expression of key genes such as adiponectin and β-klotho. This subsequently impacts fatty acid oxidation and insulin sensitivity, thereby driving the pathological progression of MASLD [50,51]. Ultimately, multiple mechanisms intertwine to form a vicious cycle, collectively driving the onset, progression, and evolution of MASLD towards metabolic dysfunction-associated steatohepatitis (MASH) and more advanced liver disease.

### 3.3. Mechanism of ALD

**Figure 3:**
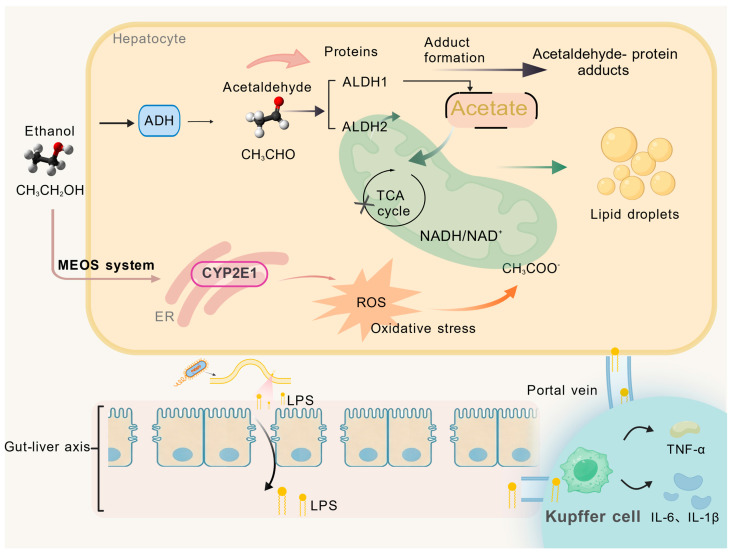
Mechanisms of ALD. Ethanol is primarily metabolized in the liver by ADH to acetaldehyde, which forms acetaldehyde-protein adducts and contributes to hepatocellular dysfunction. Acetaldehyde is further oxidized by ALDH1/ALDH2 to acetate, accompanied by an increased NADH/NAD^+^ ratio, inhibition of the TCA cycle, and lipid accumulation. Chronic alcohol intake also induces CYP2E1 via the MEOS pathway, generating excessive ROS and oxidative stress. In addition, gut barrier disruption promotes LPS translocation into the portal vein, activating Kupffer cells and triggering inflammatory cytokine release, thereby exacerbating liver injury. Abbreviations: ADH, Alcohol dehydrogenase; ALDH, Aldehyde dehydrogenase; CYP2E1, Cytochrome P450 2E1; ER, Endoplasmic reticulum; IL-1β, Interleukin-1 beta; IL-6, Interleukin-6; LPS, Lipopolysaccharide; MEOS, Microsomal ethanol-oxidizing system; NADH/NAD^+^, ratio of reduced nicotinamide adenine dinucleotide to its oxidized form; ROS, Reactive oxygen species; TCA, Tricarboxylic acid cycle; TNF-α, Tumor necrosis factor-alpha. Created with BioGDP.com [26].

ALD is one of the most common causes of liver-related morbidity and mortality worldwide and is the most prevalent form of chronic liver disease associated with excessive alcohol consumption [52]. The World Health Organization reports that 2.6 million deaths globally each year are attributable to alcohol consumption, accounting for 4.7% of all deaths [53]. ALD typically begins as fatty liver/steatosis, progressing to alcoholic hepatitis, liver fibrosis or cirrhosis [52,54]. Its pathogenesis is complex. After being consumed, it is absorbed into the blood circulation through the gastrointestinal tract, and more than 95% of absorbed alcohol is metabolized by the liver [55]. The alcohol dehydrogenase system in the liver converts ethanol into acetaldehyde, which then binds to macromolecules within the liver (such as proteins and nucleic acids) to form acetaldehyde-protein adducts [56]. These adducts alter protein function and structure, disrupting normal cellular metabolism and physiological functions, ultimately causing hepatocyte damage [57]. Meanwhile, this process raises the hepatic NADH/NAD^+^ ratio, suppresses the mitochondrial tricarboxylic acid cycle (TCA), causes fat buildup in the liver, and further facilitates the progression of fatty liver disease [58,59]. Chronic alcohol consumption significantly induces activation of the microsomal ethanol oxidizing system, upregulating the expression and activity of CYP2E1 [60]. Oxidative alcohol metabolism in hepatocytes generates reactive oxygen species, causing damage to hepatocytes [61]. Furthermore, chronic alcohol consumption disrupts the gut-liver axis, causing gut microbiota imbalance and increased barrier permeability [62,63]. This permits endotoxins (e.g., LPS) to enter the portal vein system, activating immune cells like Kupffer cells to release inflammatory mediators such as tumor necrosis factor-α (TNF-α), IL-1β, and IL-6 [64,65]. This triggers hepatic inflammation, further exacerbating liver damage [66] (Figure 3).

### 3.4. Mechanism of Viral Hepatitis

In addition to alcoholic liver damage, viral hepatitis is another major cause of liver disease worldwide. It is a systemic infectious disease characterized by liver inflammation and necrotic lesions, caused by infection with specific hepatotropic viruses. Its primary pathogens include the hepatitis A virus (HAV), hepatitis B virus (HBV), hepatitis C virus (HCV), hepatitis D virus (HDV), and hepatitis E virus (HEV). The viruses exhibit significant differences in transmission routes and pathogenic mechanisms, posing a persistent threat to global public health. The 69th World Health Assembly endorsed the Global Health Sector Strategy on Viral Hepatitis, which aims to eliminate viral hepatitis as a public health threat by 2030 [67].

#### 3.4.1. HAV and HEV

Among the hepatotropic viruses mentioned above, both HAV and HEV are transmitted via the fecal-oral route [68]. After oral ingestion, these viruses cross the intestinal mucosa, enter the bloodstream, and ultimately reach the liver. They replicate within the cytoplasm of hepatocytes and are excreted into bile via the bile ducts, re-entering the fecal-oral transmission cycle [69]. Hepatocyte injury does not primarily result from direct cytopathic destruction, but rather from immune-mediated mechanisms [70,71]. Infections with HAV and HEV are typically self-limited diseases. During this process, both innate and adaptive immune responses are involved, including the activation of NK cells, CD8^+^ T cells, and CD4^+^ T cells, which clear the virus by secreting cytokines such as interferon-γ. However, triggering an excessive host immune response can induce hepatocyte apoptosis, thereby inducing liver inflammation while eliminating the virus. Studies indicate that HAV-related liver failure is caused by an excessive host response [72]. In contrast, in HEV infection, an inadequate immune response leads to chronic infection [73]. In immunocompromised patients, antigen-specific CD4^+^ and CD8^+^ T cell responses are significantly weakened, with impaired function and reduced numbers, preventing effective viral clearance; thus, persistent viral presence ultimately results in chronic infection [73].

#### 3.4.2. HBV

HBV is a hepatotropic DNA virus transmitted primarily through intravenous drug use, sexual contact, or vertical mother-to-child transmission [71], posing a significant challenge to global public health. Following transmission, HBV enters hepatocytes through an initial low-affinity attachment to heparan sulfate proteoglycans (HSPGs), followed by high-affinity binding to sodium taurocholate cotransporting polypeptide, its functional receptor on hepatocytes [74]. After invading hepatocytes [75], it forms covalently closed circular DNA (cccDNA) in the nucleus, which serves as a stable transcriptional template that continuously drives viral replication. During infection, the HBeAg mediates immune evasion by inhibiting dendritic cell maturation and inducing regulatory T cell differentiation. Simultaneously, it activates macrophages via the TLR2/NF-κB pathway, leading to the release of inflammatory mediators such as TNF-α and IL-6, which exacerbate CD8^+^ T cell-mediated hepatocellular damage [76]. Furthermore, HBV infection induces gut microbiota dysbiosis and disrupts intestinal barrier function, allowing microbial products such as LPS to enter the liver via the portal vein. This activates Kupffer cells, exacerbating systemic inflammation and promoting the progression of liver injury [77,78,79]. Notably, HBV infection may alter gut microbiota composition and impair intestinal barrier function, thereby contributing to disease progression [80].

#### 3.4.3. HCV

HCV is a single-stranded, quasi-enveloped RNA virus [71] primarily transmitted through blood and constitutes a major risk factor for cirrhosis and hepatocellular carcinoma (HCC) [81]. HCV binds to host cell surface receptors (e.g., CD81, SR-BI) via its envelope glycoproteins E1 and E2 and enters cells through clathrin-dependent endocytosis [82,83]. HCV replication is closely associated with the ER. Its protein expression induces ER stress, which activates the unfolded protein response and promotes hepatocyte apoptosis through pathways such as PERK-eIF2α-CHOP and may also induce autophagy [84]. Additionally, for immune evasion, the E2 protein of HCV continuously accumulates mutations, enabling it to effectively escape broadly neutralizing antibodies (bNAbs, also known as neutralizing antibodies with broad spectrum) produced by the host. This immune evasion mechanism enables HCV to establish and sustain chronic infection [85].

#### 3.4.4. HDV

HDV is a defective satellite RNA virus [86]. The assembly, infectivity, and propagation of HDV depend on HBsAg, whereas its genomic replication relies primarily on host cellular enzymatic machinery [71,87]. HDV entry into hepatocytes is similar to that of HBV, followed by translocation of the viral ribonucleoprotein complex into the nucleus. Within the nucleus, HDV utilizes its own genome to replicate via a rolling circle replication (RCR) mechanism [88]. Research indicates that HDV possesses direct cytopathic effects. During the acute infection phase, hepatocytes exhibit degenerative changes such as cytoplasmic eosinophilia and nuclear condensation, accompanied by relatively mild inflammatory cell infiltration. This finding suggests that the virus itself may directly mediate hepatocyte damage [89]. Chronic HDV infection typically leads to rapid progression of liver disease, manifesting as significant fibrosis, cirrhosis, and a markedly increased risk of hepatocellular carcinoma (HCC) [90].

Taken together, the five types of hepatitis viruses differ substantially in their core virological features, including nucleic acid structure, route of infection, and mechanisms of entry and replication. A detailed comparison of these key characteristics is systematically summarized in Table 2.

### 3.5. Mechanism of AILD

AILD refers to a class of liver disorders primarily characterized by liver damage and abnormal liver function, which are caused by abnormal immune system responses. It primarily includes primary biliary cholangitis, autoimmune hepatitis (AIH), primary sclerosing cholangitis (PSC), and immunoglobulin G4-related cholangitis [91]. As research into AILD deepens, its pathogenesis has been found to be primarily associated with genetic predisposition, environmental factors, immune activation, and macrophage programmed cell death [92].

Regarding genetic predisposition, AIH can occur in individuals of all ages, both sexes, and all ethnic groups. One third of patients have already developed cirrhosis at the moment of diagnosis, suggesting a delay in diagnosis. AIH develops in genetically predisposed individuals, after exposure to triggering factors like microbes, viruses or drugs [93]. Studies have shown that genetic predisposition to AIH is primarily associated with human leucocyte antigen genes, with the human leukocyte antigen region being the most significant susceptibility locus for AIH-1 [94].

Regarding environmental factors, intestinal microbial dysbiosis acts as a key trigger. Disrupted intestinal flora compromises the mucosal barrier, allowing LPS to enter the liver via the portal vein. Kupffer cells in the liver recognize LPS, activating signaling pathways like NF-κB and triggering massive release of TNF-α and IL-1 [95]. These pro-inflammatory factors directly induce hepatocyte injury or death by activating pathways like JNK. Simultaneously, they create an inflammatory microenvironment that promotes the activation, proliferation, and differentiation of CD8^+^ T cells into cytotoxic T lymphocytes. These activated immune cells further secrete inflammatory mediators, forming a vicious cycle of immune-mediated injury, which ultimately leads to persistent liver tissue damage, driving the progression of AILD [96]. Concurrently, dysregulated immune activation plays a central role in AILD pathogenesis. When hepatocytes are damaged, intracellular self-antigens, such as cytochrome P450 2D6 (CYP2D6) become exposed on the cell surface. These antigens are captured by antigen-presenting cells and presented to CD4^+^ T cells, disrupting immune tolerance and activating autoreactive T cell clones. Activated CD4^+^ T cells subsequently differentiate into distinct Th cell subsets that synergistically mediate liver injury: Th1 cells activate CD8^+^ cytotoxic T cells by secreting interferon-γ (IFN-γ) leading to direct hepatocyte lysis; Th2 cells assist B cells in producing pathogenic autoantibodies; while Th17 cells exacerbate local inflammation and fibrosis by secreting cytokines such as IL-17. Concurrently, a reduction in the number or functional impairment of regulatory T cells, which possess immunosuppressive functions, prevents effective termination of the immune attack, leading to persistent inflammation and liver damage [96,97].

**Figure 4:**
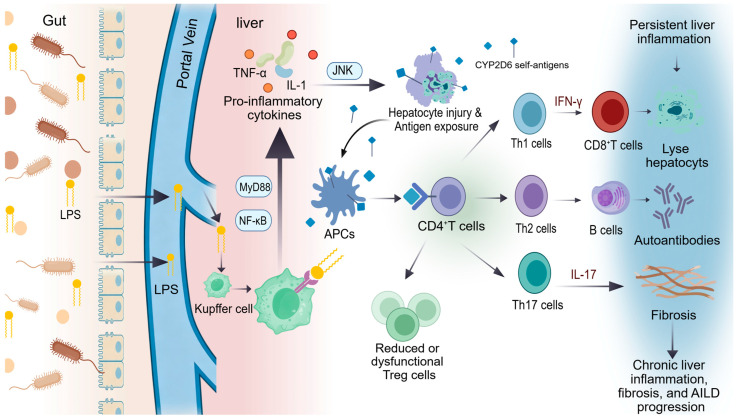
Mechanisms of AILD. Intestinal dysbiosis impairs the gut barrier and facilitates LPS translocation to the liver via the portal vein, where it activates Kupffer cells and downstream NF-κB/MyD88 signaling, leading to the release of pro-inflammatory cytokines, including TNF-α and IL-1. These mediators induce hepatocyte injury through JNK activation and promote self-antigen exposure (e.g., CYP2D6), which is captured by APCs and presented to CD4^+^ T cells. Subsequently, Th1, Th2, and Th17 responses, together with CD8^+^ T cell cytotoxicity, autoantibody production, and impaired Treg-mediated immune suppression, amplify hepatic inflammation, fibrosis, and ultimately drive AILD progression. Abbreviations: AILD, Autoimmune liver disease; APCs, Antigen-presenting cells; CD8^+^ T, Cluster of differentiation 8-positive T lymphocyte; CD4^+^ T, Cluster of differentiation 4-positive T lymphocyte; CYP2D6, Cytochrome P450 2D6; IFN-γ, Interferon-γ; IL-1, Interleukin-1; IL-17, Interleukin-17; JNK, C-Jun N-terminal kinase; LPS, Lipopolysaccharide; MyD88, Myeloid differentiation primary response 88; NF-κB, Nuclear factor κ B; Th, T helper; TNF-α, Tumor necrosis factor-α; Treg, Regulatory T. Created with BioGDP.com [23].

Overall, AILD arises from the combined effects of genetic predisposition, environmental insults, and loss of immune tolerance. Susceptibility genes, particularly within the human leukocyte antigen region, interact with triggers such as infections, drugs, and intestinal microbial dysbiosis to initiate hepatic inflammation. Subsequent activation of innate and adaptive immune responses, together with impaired regulatory T-cell function, sustains chronic immune-mediated hepatocyte injury and promotes disease progression. A schematic overview of these pathogenic mechanisms is presented in Figure 4.

In AIH, macrophages are increasingly recognized as important contributors to hepatic inflammation and disease progression [98]. Recent experimental evidence indicates that dysregulated macrophage polarization is involved in AIH pathogenesis. In particular, pro-inflammatory M1 macrophage polarization has been associated with aggravated liver injury, whereas suppression of M1 polarization attenuates hepatic inflammation in experimental AIH [99]. In addition, monocyte-derived macrophages have been shown to exacerbate immune-mediated liver injury, further supporting the role of inflammatory macrophage subsets in disease progression [100]. Epigenetic regulation also contributes to this process, as EZH2-mediated H3K27me3 promotes AIH progression by modulating macrophage polarization [101]. Together, these findings support macrophage polarization as a relevant mechanistic component and a potential therapeutic target in AIH.

## 4. Mechanisms of the Hepatoprotective Effects of Natural Alkaloids

### 4.1. DILI

Natural alkaloids have gained significant attention in recent years for their potential in preventing and treating DILI. Extensive studies have been conducted to elucidate their protective effects against DILI, molecular targets, and underlying signaling pathways, generating substantial evidence. Depending on their distinct therapeutic targets, these alkaloids mediate hepatoprotection against DILI through multiple mechanisms, including the modulation of oxidative stress, inflammatory responses, and hepatocyte apoptosis.

**Figure 5:**
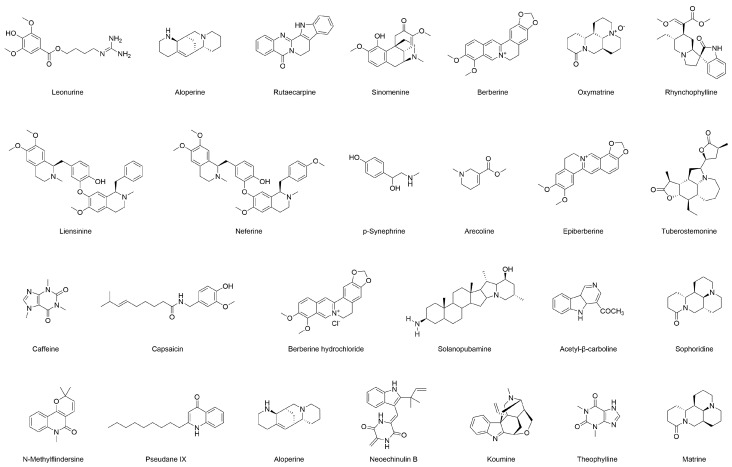
Schematic diagram of alkaloid monomers with hepatoprotective effects. (Sangzhi alkaloids (SZ-A) are a complex mixture of polyhydroxy alkaloids extracted from *Morus alba* L. (mulberry twig). They are not a single compound and therefore do not have a defined chemical structure.)

Due to its defined dose-dependent hepatotoxicity and well-elucidated molecular mechanisms, APAP is the most widely adopted model drug in this area of study. For example, a study by Yajie Yu et al. [102] found that Leonurine, a natural alkaloid isolated from the herb *Leonurus japonicus* Houtt, can alleviate APAP-induced acute liver injury in mice by reducing serum ALT/AST levels, alleviating liver histopathological damage, and mitigating hepatocyte necrosis and oxidative stress. Leonurine exerts its hepatoprotective effects by activating the Nrf2 pathway, enhancing antioxidant enzyme expression, and reducing oxidative damage. Additionally, Leonurine inhibits liver inflammation by suppressing the TLR4 and NLRP3 pathways. The PI3K/AKT signaling pathway was identified as a key regulator of Leonurine’s effects, with molecular docking and cellular thermal shift assay confirming that PI3K is a direct target of Leonurine. These findings suggest that Leonurine modulates multiple pathways, including apoptosis, oxidative stress, and inflammation, to attenuate APAP-induced liver injury. Research by Hui Chen et al. [103] revealed that Aloperine, a matrine-type alkaloid isolated from *Sophora alopecuroides* L., ameliorates APAP-induced acute liver injury by inhibiting the activation of the HMGB1/TLR4/NF-κB signaling pathway. This inhibition downregulates the release of HMGB1, the expression of TLR4, and the phosphorylation of NF-κB (p-p65) in liver tissue, reducing the production of pro-inflammatory cytokines TNF-α and IL-1β. Concurrently, it blocks NLRP3 inflammasome activation, thereby reducing NLRP3 expression and caspase-1 cleavage. Furthermore, a study by Jae Ho Choi et al. [104] demonstrated that rutaecarpine, isolated from the unripe fruit of *Evodia rutaecarpa*, significantly alleviates APAP-induced acute liver injury in mice. Rutaecarpine pretreatment significantly mitigated APAP-induced liver injury by reducing serum ALT/AST activities and hepatic malondialdehyde levels, while preserving glutathione content. This protective effect was partly due to dose-dependent inhibition of CYP2E1 expression, which plays a key role in APAP metabolism. Additionally, rutaecarpine inhibited inflammatory cytokine expression by blocking NF-κB activation through the JNK1/2 signaling pathway. Another study [105] indicated that Sinomenine alleviates oxidative stress-induced liver damage by reducing serum ALT activity and hepatic malondialdehyde (MDA) levels, while increasing GSH content to bolster endogenous antioxidant defense. It also prevents liver injury progression through mechanisms including the inhibition of hepatocyte apoptosis and modulation of inflammatory responses. Additionally, sinomenine exerts its protective effects by up-regulating the expression of SIRT1. In the APAP model, berberine protects the liver by enhancing phase II detoxification, suppressing oxidative stress, reducing hepatocyte necrosis, and inhibiting inflammatory responses. Mechanistically, these effects are achieved by activating the Nrf2/Mn-SOD signaling pathway; inhibiting JNK phosphorylation; up-regulating the expression of UGTs and SULTs; and down-regulating the expression of HMGB1, p-p65, and cleaved caspase-1 [106]. Berberine (BBR) has also demonstrated protective potential in other models of drug-induced liver injury. For instance, it has been shown to protect against methotrexate-induced hepatotoxicity by attenuating oxidative stress and apoptosis, likely through upregulation of the Nrf2/HO-1 pathway and PPARγ [107]. Furthermore, in a study by Xueyan Chen et al., berberine ameliorated doxorubicin-induced acute hepatorenal toxicity by mitigating the perturbations in the levels of oxidative stress markers, including MDA, SOD, and GPx [108]. The hepatoprotective profile of berberine also extends to bortezomib-induced injury, primarily through a multi-targeted suppression of the bortezomib-triggered expression of pro-inflammatory (NF-κB, TLR-4, TNF-α, IL-1β) and pro-apoptotic (P53, Apaf-1, Bax, Caspase-3/6/9) mediators [108,109]. Collectively, studies demonstrate berberine’s broad hepatoprotective efficacy against injuries induced by diverse agents, including, as shown in the current study, cyclophosphamide (via antioxidant potentiation and modulation of TNF-α/COX-2) [110]. Beyond the aforementioned compounds, alkaloids also exhibit hepatoprotective properties in anti-tuberculosis treatment models, with dendrobine attenuating isoniazid and rifampicin-induced liver injury in mice via miR-295-5p-dependent regulation of CYP1A2 expression [111] (Table 3, Figure 5).

Collectively, accumulating evidence demonstrates that natural alkaloids exert robust hepatoprotective effects against drug-induced liver injury, particularly APAP-induced acute liver injury, through multi-target and multi-pathway regulation. These compounds consistently alleviate hepatocellular damage by enhancing antioxidant defenses most prominently via activation of the Nrf2 signaling axis while concurrently suppressing inflammatory responses mediated by TLR4/NF-κB and NLRP3 inflammasome pathways. In addition, several alkaloids modulate drug-metabolizing enzymes and key survival pathways, such as CYP450 enzymes and PI3K/AKT signaling, thereby integrating anti-oxidative, anti-inflammatory, and anti-apoptotic effects. Importantly, the hepatoprotective efficacy of alkaloids extends beyond APAP to diverse hepatotoxic agents, underscoring their broad therapeutic potential and positioning them as promising candidates for the prevention and treatment of drug-induced liver injury.

### 4.2. MASLD

Natural alkaloids have demonstrated significant potential in the intervention of metabolic dysfunction-associated steatotic liver disease (MASLD) due to their multi-target, multi-pathway modes of action. Research indicates that these compounds primarily mitigate hepatic lipid accumulation, steatosis, and fibrosis progression through synergistic mechanisms. These include modulating core metabolic signalling pathways, enhancing mitochondrial function, suppressing inflammation and oxidative stress, and remodelling the gut microbiota.

Regarding the regulation of core metabolic pathways, BBR alleviates metabolic disorders through multiple targets, including inhibition of mitochondrial complex I to reduce ATP synthesis and down-regulate SCD1, FABP1, CD36, and CPT1A; activation of the ERK1/2 signaling pathway to up-regulate LDLR, ABCA1, ABCG1, and SR-B1 while down-regulating PCSK9, thus enhancing lipid clearance; and activation of the PI3K/AKT signaling pathway together with inhibition of the STING signaling pathway to improve insulin sensitivity and suppress inflammation [112,113,114]. Oxymatrine integrates multiple mechanisms, including the activation of the PI3K/AKT signaling pathway and up-regulation of PPARα, ACOX1, and CPT1A to enhance fatty acid oxidation, along with improving lipid metabolic disorders and modulating amino acid and bile acid metabolism, as well as up-regulating SOD, CAT, and GSH-Px while down-regulating MDA, NLRP3, IL-1β, TGF-β1, and Collagen I, thereby synergistically reducing lipid deposition and fibrosis [115,116,117]. Similarly, Rhynchophylline promotes lipid decomposition by activating hepatic lipase (Lipc) activity and lipolysis, enhances mitochondrial energy metabolism and glucose metabolism, and inhibits triglyceride accumulation and oxidative stress, thereby alleviating metabolic disorders [118].

Targeting AMPK as a central node represents another pivotal strategy. Liensinine acts by activating the TAK1/AMPK signaling pathway to correct lipid dysregulation while concurrently inhibiting the TAK1/NF-κB signaling pathway to suppress NF-κB-mediated inflammation [119]. Its analog, Neferine, activates the AMPK/acetyl-CoA carboxylase (ACC) signaling pathway while inhibiting the TLR4/NF-κB p65 and TGFβ-Smad2/3 signaling pathways [120]. Furthermore, Sangzhi alkaloids inhibit hepatic lipid synthesis by activating the AMPK/ACC pathway and improve systemic metabolism by upregulating the expression of PPARα and PGC-1α [121]. *p*-Synephrine ameliorates HFD-induced MASLD by activating AMPK, inhibiting hepatic de novo lipogenesis through downregulation of SREBP-1c, FASN, and ACC1, improving insulin signaling via IRS-1, PI3K, AKT, and GLUT-4, suppressing NF-κB-mediated inflammation by reducing TNF-α, IL-6, and IL-1β, and promoting adipose tissue browning via UCP1 and PGC-1α, thereby regulating the liver-adipose axis through the AMPK/NF-κB pathway [122].

Concerning the gut-liver axis, arecoline ameliorates host lipid metabolic disorders indirectly by reshaping the gut microbiota composition, promoting the proliferation of beneficial bacteria, and reprogramming microbial metabolic functions, providing a rationale for microbiota-metabolism axis-based interventions [123]. Epiberberine ameliorates hepatic steatosis by upregulating small heterodimer partner (SHP), which in turn inhibits the SREBP-1c/FASN signaling pathway to reduce triglyceride synthesis, while also improving gut microbiota homeostasis [124].

Other alkaloids ameliorate MASLD through distinct mechanisms. Tuberostemonine alleviates MASLD by inhibiting fat deposition, reducing oxidative stress, increasing ATP and mitochondrial membrane potential, and improving glucose and energy metabolism, thereby reducing weight gain and fat accumulation [125]. Caffeine ameliorates NASH by repressing the AKT/mTORC1 and SREBP-1/ACC/FASN signaling pathways to inhibit hepatic de novo lipogenesis, while also impairing NF-κB activation via IκBα stabilization to reduce IL-6 and TNF-α, and abolishing mTORC1/FASN-dependent MyD88 palmitoylation to suppress inflammation [126]. (Table 4, Figure 5).

In summary, natural alkaloids intervene in the complex pathological network of MASLD/NAFLD in a pleiotropic manner by acting on multiple key nodes such as AMPK, PPARα, gut microbiota, and inflammatory signaling networks. These studies not only deepen the understanding of the disease mechanisms but also lay a crucial preclinical foundation for developing novel multi-target therapeutic strategies based on natural products.

### 4.3. ALD

In the study of interventions against alcoholic liver injury, natural alkaloids have garnered significant attention due to their ability to simultaneously target multiple key steps in disease progression. Current evidence indicates that such compounds can significantly mitigate ethanol-induced liver damage by coordinately regulating core pathological processes including oxidative stress, inflammatory response, dysregulated lipid metabolism, and cell death.

Capsaicin consistently exhibited hepatoprotective effects against alcohol-induced liver injury through coordinated regulation of oxidative stress, inflammatory signaling, fibrogenesis, and lipid metabolism. Mechanistically, capsaicin suppressed ethanol-induced cytochrome P450 2E1 (CYP2E1) activity, resulting in reduced reactive oxygen species production, preservation of mitochondrial function and membrane potential, and attenuation of oxidative damage [127]. In parallel, capsaicin markedly inhibited nuclear factor kappa B (NF-κB) activation and downregulated pro-inflammatory mediators, including tumor necrosis factor-α and interleukin-6, thereby alleviating hepatic inflammatory responses [127]. Furthermore, capsaicin exerted anti-fibrotic effects by restoring the balance between matrix metalloproteinases and tissue inhibitors of metalloproteinases, suppressing extracellular matrix accumulation and collagen deposition, and directly inhibiting hepatic stellate cell activation [127,128]. capsaicin ameliorates alcohol-induced liver injury primarily by exerting antioxidant effects, especially through reducing hepatic lipid peroxidation and oxidative stress. [129].

Zhang et al. reported that berberine exerts hepatoprotective effects against alcohol-induced liver injury primarily through coordinated attenuation of oxidative stress and hepatic steatosis. Mechanistically, berberine suppresses oxidative stress by downregulating both total and mitochondria-localized cytochrome P450 2E1 (CYP2E1). This reduction alleviates ethanol-induced mitochondrial lipid peroxidation, glutathione depletion, and mitochondrial swelling. In parallel, berberine ameliorates alcohol-induced lipid accumulation by restoring the expression of peroxisome proliferator-activated receptor α (PPARα) and its coactivator PGC-1α, thereby enhancing fatty acid β-oxidation via target genes such as CPT1α, ACO, and MCAD. Berberine also upregulates hepatocyte nuclear factor 4α (HNF4α) and mTTP to promote hepatic lipid export. These combined effects mitigate steatosis and hepatocellular damage without disturbing normal liver homeostasis [130].

Furthermore, research by Hui Fan et al. indicates that sinomenine can significantly alleviate alcoholic liver injury by coordinately modulating the Nrf2/HO-1 antioxidant pathway, the NF-κB/AKT inflammatory pathway, and the JNK/p38 MAPK apoptotic pathway [131] (Table 5, Figure 5).

In summary, natural alkaloids such as capsaicin, berberine, and sinomenine demonstrate clear protective effects in alcoholic liver injury models by regulating multiple pathways including oxidative stress, inflammatory response, lipid metabolism, and cell apoptosis. This provides an experimental foundation for their further development as anti-alcoholic liver disease agents.

### 4.4. Viral Hepatitis

Research indicates that the potential of alkaloids in the antiviral field, particularly for the treatment of viral hepatitis, is receiving increasing attention. Studies have shown that various alkaloids can inhibit the replication and infection processes of HBV and HCV through different mechanisms, with some compounds also possessing hepatoprotective effects, demonstrating the advantage of multi-target therapy.

In terms of anti-HBV effects, several alkaloids have demonstrated well-defined mechanisms of action. A study by Mohammad K. Parvez et al. found that solanopubamine, a rare steroidal alkaloid isolated from the *Solanum schimperianum* plant, not only inhibits both wild-type and lamivudine-resistant mutant HBV polymerase but also alleviates oxidative stress and downregulates caspase-3/7 activity, thereby exhibiting both antiviral and hepatoprotective functions [132]. Similarly, the alkaloid acetyl-β-carboline from the aerial parts of *Rhazya stricta* has also been confirmed to directly target HBV polymerase (including drug-resistant mutants), inhibiting HBV antigen secretion in a dose- and time-dependent manner, thus exerting in vitro anti-HBV activity [133]. Additionally, berberine exerts anti-HBV effects by enhancing TRIM21-mediated K^48^-linked polyubiquitination and subsequent proteasomal degradation of the HNF4α protein, thereby inhibiting HBV core promoter activity and blocking HBV replication and cccDNA synthesis [134]. Sophoridine inhibits viral replication by downregulating the expression of HBsAg, HBeAg, p38MAPK, TRAF6, ERK1, NLRP10 and Caspase-1, thereby promoting HBV DNA methylation [135]. Matrine, the primary active alkaloid extracted from *Sophora flavescens*, exerts anti-HBV effects through multiple pathways: directly inhibiting viral DNA synthesis and transcription to reduce viral load; inducing endogenous interferon production and modulating immunity to suppress inflammatory factor release; and blocking hepatic fibrogenesis while promoting hepatocyte repair [136].

In anti-HCV research, alkaloids also exhibit diverse mechanisms of action. For instance, N-methylflindersine, isolated from the fruits of *Melicope latifolia* targets the post-entry phase of the viral replication cycle. It inhibits NS3 protein expression in a dose-dependent manner, interferes with viral assembly, and its quinoline structure may synergistically block NS5B RNA polymerase function, thereby exerting potent anti-HCV activity [137]. Pseudane IX, isolated from the dichloromethane extract of *Ruta angustifolia* leaves, inhibits HCV at the post-entry stage, reducing viral RNA replication and downregulating the expression of HCV NS3 [138]. Aloperine interferes with the process from viral endocytosis to membrane fusion, simultaneously inhibiting HCV entry, replication, and cell-to-cell spread [139]. In addition to its anti-HBV activity, berberine can also achieve potent and low-toxicity anti-HCV activity by specifically binding to the HCV E2 glycoprotein, thereby blocking viral attachment and the entry/fusion process [140].

Furthermore, the fungus-derived alkaloid neoechinulin B inhibits HCV proliferation by targeting the host liver X receptor and disrupting the lipid metabolism-dependent viral replication vesicles, demonstrating highly selective antiviral properties [141] (Table 6, Figure 5).

### 4.5. AILD

Natural alkaloids also demonstrate the potential for multi-target and multi-pathway regulation in the treatment of autoimmune hepatitis (AIH). Research indicates that various alkaloids can effectively alleviate Concanavalin A (Con A)-induced liver injury by modulating key signaling pathways and immune-inflammatory responses.

Koumine, a principal alkaloid from *Gelsemium elegans*, alleviates Con A-induced autoimmune hepatitis in mice by activating the Nrf2/HO-1 signaling pathway, up-regulating the expression of Bcl-2, and down-regulating the expression of Keap-1, IL-6, TNF-α, NF-κB, TLR4, TRAF6, and Bax [142].

BBR, by activating the AMPK signaling pathway, reduces serum transaminase levels, inhibits hepatocyte swelling and necrosis, and downregulates inflammatory factors such as TNF-α, IFN-γ and IL-2, thereby significantly alleviating Con A-induced AIH-related liver injury [143].

Notably, matrine alleviates Con A-induced immune-mediated liver injury through a synergistic mechanism encompassing anti-inflammatory, anti-fibrotic, and anti-apoptotic effects. It reduces serum IFN-γ, hepatic TNF-α and IL-6 levels, attenuates mononuclear cell infiltration, inhibits collagen deposition and hepatic hydroxyproline content, and downregulates caspase-3 expression to reduce hepatocyte apoptosis [144]. Mechanistically, matrine coordinately modulates multiple signaling pathways, including NF-κB, JNK, JAK1/STAT3, and PI3K/AKT, thereby suppressing inflammatory responses, oxidative stress, and hepatocyte apoptosis [145].

Natural alkaloids exhibit multi-target and multi-pathway therapeutic potential against autoimmune hepatitis (AIH). As demonstrated in Concanavalin A (Con A)-induced liver injury models, alkaloids such as koumine, BBR, and matrine alleviate hepatic damage through coordinated regulation of inflammatory, apoptotic, and fibrotic processes. Koumine activates the Nrf2/HO-1 antioxidant pathway and modulates Bcl-2/Bax balance while suppressing NF-κB-mediated inflammation. BBR engages the AMPK signaling pathway to reduce transaminase levels, inhibit hepatocyte necrosis, and downregulate pro-inflammatory cytokines (TNF-α, IFN-γ, IL-2). Matrine exerts synergistic anti-inflammatory, anti-fibrotic, and anti-apoptotic effects by modulating NF-κB, JNK, JAK1/STAT3, and PI3K/AKT pathways, thereby reducing inflammatory infiltration, collagen deposition, and caspase-3-dependent hepatocyte apoptosis. Collectively, these findings highlight the promising role of natural alkaloids as multi-target regulators in the treatment of AIH (Table 7, Figure 5).

### 4.6. Current Effectiveness and Translational Limitations of Alkaloid-Based Hepatoprotection

Although the alkaloids discussed above consistently show hepatoprotective or antiviral activities in experimental models, their apparent effectiveness should be interpreted with caution. The current evidence is largely derived from in vitro assays and animal models, and the efficacy of different alkaloids cannot be directly compared because of differences in disease models, doses, routes of administration, intervention timing, endpoints, and experimental designs. Among the compounds reviewed, berberine has relatively broad and reproducible evidence across several liver injury settings, including DILI, MASLD, ALD, viral hepatitis, and AIH-related models, mainly through the regulation of oxidative stress, inflammation, lipid metabolism, and immune signaling. Other alkaloids appear to have more condition-specific advantages; for example, capsaicin and sinomenine show notable effects in alcohol-related or inflammatory liver injury models, matrine and koumine are mainly supported by immune-mediated hepatitis models, whereas several antiviral alkaloids have so far been evaluated predominantly in cell-based HBV or HCV systems.

This distinction becomes even more evident when their clinical development and approved indications are considered. Among the alkaloids listed, oxymatrine and matrine are currently the most clearly established representatives that have been approved and clinically used in China for liver-related diseases, particularly chronic viral hepatitis. Both compounds have been approved by the National Medical Products Administration of China for use in chronic viral hepatitis and as adjunctive therapy for liver fibrosis. Sophoridine has also been used clinically, mainly as an adjunctive treatment for malignant tumors, including primary liver cancer. By contrast, although berberine has accumulated substantial clinical evidence in areas such as MASLD, it has not yet been approved for liver-related indications. Most of the other alkaloids remain at the basic research stage, or, although registered for clinical use, their approved indications are unrelated to liver diseases (Table 8).

Therefore, these compounds should currently be regarded primarily as promising lead compounds or adjunctive therapeutic candidates rather than universally established clinical therapies for liver diseases. The continued global burden of DILI, MASLD, ALD, viral hepatitis, and autoimmune liver diseases reflects several translational barriers, including limited clinical validation, uncertain pharmacokinetic properties, poor or variable bioavailability, potential toxicity or drug–drug interactions, disease heterogeneity, and the lack of large-scale randomized controlled trials. Future studies should prioritize standardized efficacy comparisons, dose–response analyses, safety evaluation, pharmacokinetic optimization, and well-designed clinical trials to determine which alkaloids have genuine therapeutic value in human liver diseases.

## 5. Conclusions and Future Perspectives

In conclusion, the evidence synthesized in this review indicates that liver injury, although initiated by diverse etiologies including DILI, MASLD, ALD, viral hepatitis, and AILD, follows a set of deeply interconnected molecular trajectories. Oxidative stress, inflammatory activation, hepatocyte apoptosis, lipid metabolic disturbance, inflammasome signaling, and mitochondrial dysfunction do not operate in isolation; rather, they interact dynamically to shape hepatic damage and to promote progression toward fibrosis, cirrhosis, and ultimately, liver failure. Against this background, natural alkaloids stand out not because they act on a single dominant target but because they appear capable of modulating several critical pathogenic nodes simultaneously. Across the studies discussed in this review, recurrent involvement of the Nrf2/ARE, NF-κB, and PI3K/AKT pathways suggests that the hepatoprotective activity of alkaloids is closely related to their capacity to restore redox balance, dampen inflammatory signaling, and preserve hepatocellular homeostasis under conditions of sustained injury.

This raises an important point for the field: if liver injury is fundamentally multifactorial, should its pharmacological management not also be interpreted through a multi-target framework? While the current literature largely supports this perspective, it also underscores that mechanistic understanding remains incomplete. In particular, the relative contribution of shared versus etiology-specific pathways has not been fully delineated, and the extent to which individual alkaloids converge on common molecular networks or operate through distinct regulatory patterns requires further clarification. Future studies should therefore prioritize mechanism-oriented and comparative analyses that integrate signaling events with specific pathological contexts, thereby strengthening the biological rationale for alkaloid-based hepatoprotective strategies and clarifying their translational relevance.

Despite encouraging preclinical evidence, the translational application of alkaloids still faces multiple challenges. Future research should focus on the identification of molecular targets and structure–activity relationships, as well as the optimization of pharmacokinetic properties through advanced delivery strategies. Meanwhile, mechanism-oriented and comparative studies are needed to improve reproducibility and strengthen the linkage between molecular mechanisms and pathological contexts. In addition, biomarker-driven approaches and rational combination strategies may further enhance therapeutic efficacy and promote the development of alkaloids as hepatoprotective agents.

## Figures and Tables

**Table 1:** Mechanisms of common drugs induced DILI.

Drug Name	Molecular Structure	Indication/Drug Action	Mechanism of Hepatotoxicity	Refs.
APAP	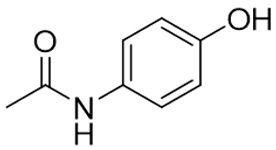	Non-opioid analgesic	NAPQI protein adduct formation and oxidative stress	[18]
Isoniazid	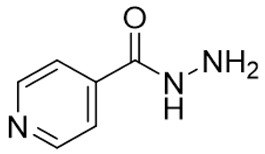	Anti-tubercular	Reactive metabolite formation, oxidative stress and mitochondrial injury	[27]
Diclofenac	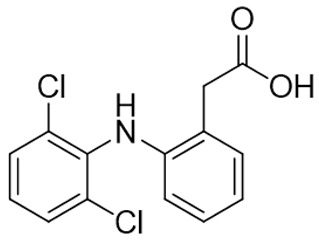	Nonsteroidal anti-inflammatory drug	Reactive metabolite formation, mitochondrial dysfunction and oxidative stress	[13,28]
Rifampicin	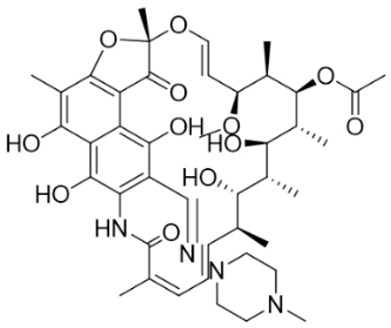	Antibiotic	Cholestasis endoplasmic and reticulum stress	[29]
Atorvastatin	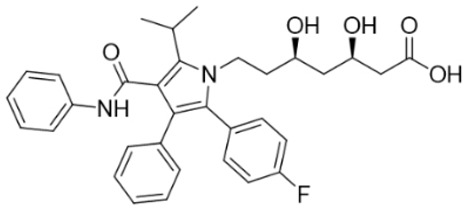	3-Hydroxy-3-methylglutaryl-coenzyme A reductase inhibitor	Mitochondrial dysfunction, oxidative stress and bile acid transporter dysregulation	[13,30]
Oxaliplatin	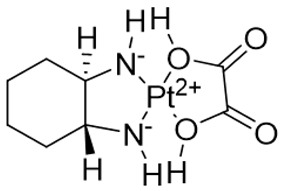	Conventional chemotherapy drugs	Damage to HSECs, oxidative stress and lipid metabolism disorder	[16,31]
Methotrexate	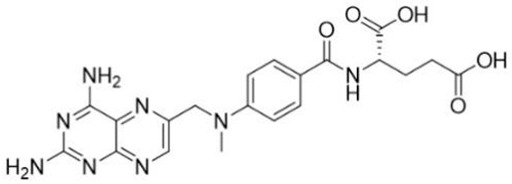	Conventional chemotherapy drugs	Oxidative stress, inflammation and steatosis/steatohepatitis	[32]
Cyclophosphamide	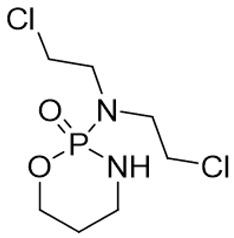	Conventional chemotherapy drugs	Reactive metabolite formation, oxidative stress, inflammation and mitochondrial dysfunction	[33]
Crizotinib	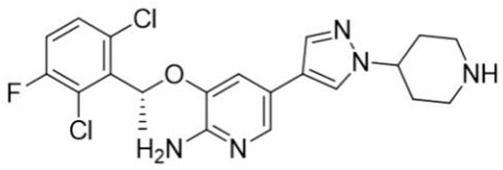	Molecular targeted agents	ROS generation, mitochondrial dysfunction and DNA damage	[34]
Tamoxifen	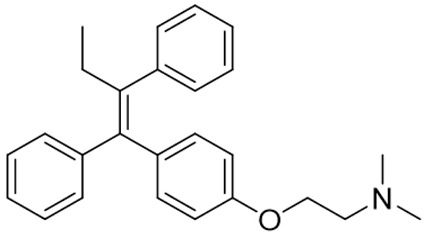	Nonsteroidal anti-estrogen	Cholestasis/bile acid transport disturbance and mitochondrial dysfunction	[32,36]

**Table 2:** Features of Hepatitis Viruses.

Virus	Nucleic Acid Structure	Route of Infection	Entry and Replication Mechanisms	Refs.
HAV	Positive-sense, single-stranded RNA	Fecal-oral route	HAV crosses the intestinal mucosa, enters the bloodstream, reaches the liver, infects hepatocytes, replicates in the cytoplasm, and is excreted in bile.	[68,69,70,71,72]
HBV	Circular, double-stranded DNA with single-stranded portions	Blood, sexual, and parenteral	HBV initially binds to hepatocytes with low affinity via heparan sulfate proteoglycans and subsequently with high affinity via sodium taurocholate cotransporting polypeptide. After entry, it forms nuclear cccDNA, which serves as a stable transcriptional template for persistent viral replication.	[71,74,75,76]
HCV	Positive-sense, single-stranded RNA	Blood, sexual, and parenteral	HCV binds to host cell receptors and enters cells via clathrin-mediated endocytosis.	[71,81,82,83,84,85]
HDV	Negative-stranded circular RNA	Blood, sexual, and parenteral	HDV enters hepatocytes through HBsAg-mediated binding to NTCP, followed by nuclear import of its circular RNA genome and host RNA polymerase II-dependent rolling-circle replication.	[71,86,87,88]
HEV	Positive sense, single-stranded RNA	Fecal-oral	Similar to HAV, HEV crosses the intestinal mucosa, enters the circulation, reaches the liver, replicates in hepatocyte cytoplasm, and is excreted via bile.	[68,69,70,71,72,73]

**Table 3:** Mechanisms of Alkaloids against DILI.

Compound	In Vivo or In Vitro Models	Mechanism of Action	Ref.
Leonurine	APAP-induced primary hepatocytes; APAP-induced acute liver injury ICR mice model	Activating the Nrf2 and PI3K/AKT signaling pathways; inhibiting TLR4 and NLRP3 signaling pathways	[102]
Aloperine	APAP-induced acute liver injury BALB/c mice model	Inhibiting the HMGB1/TLR4/NF-κB and NLRP3/inflammasome signaling pathways	[103]
Rutaecarpine	APAP-induced acute liver injury ICR mice model	Inhibiting the JNK1/2 and NF-κB signaling pathways; down-regulation of the expression of Nrf2, GCLC, HO-1, and NQO1	[104]
Sinomenine	APAP-induced liver injury Wistar rat model	Lowering the level of ALT, MDA and GSH; up-regulation of the expression of SIRT1	[105]
Berberin	APAP-induced liver injury C57BL/6 mice model	Activating the Nrf2/Mn-SOD signaling pathway; inhibiting JNK-phosphorylation; up-regulation of the expression of UGTs and SULTs; down-regulation of the expression of HMGB1, p-p65 and cleaved caspase-1	[106]
	Methotrexate-induced liver injury Wistar rat model	Activating the Nrf2/HO-1 signaling pathway; up-regulation of the expression PPARγ	[107]
	Doxorubicin-induced acute liver injury SD rats model	Up-regulation of the expression of CAT, SOD and GPx; down-regulation of the expression of MDA.	[108]
	Bortezomib-induced liver injury SD rats model	Down-regulation of the expression of NF-κB, TLR-4, TNF-α, IL-1β, P53, Apaf-1, Bax, and Caspase-3/6/9	[109]
	Cyclophosphamide-induced liver injury Albino rats model	Up-regulation of the expression of CSH, SOD and GPx; down-regulation of the expression of TNF-α and COX-2	[110]

**Table 4:** Mechanisms of Alkaloids against MASLD.

Compound	In Vivo or In Vitro Models	Mechanism of Action	Ref.
Berberine	Oleic acid (OA)-induced lipid accumulation in primary hepatocytes; high-fat-diet (HFD)-induced hepatic steatosis C57BL/6J mice model	Inhibiting mitochondrial complex I; down-regulation of the expression of SCD1, FABP1, CD36, and CPT1A.	[112]
	HFD-induced ApoE^−/−^ mice model; HFD-induced C57BL/6J mice model	Activating the ERK1/2 signaling pathway; up-regulation of the expression of LDLR, ABCA1, ABCG1, and SR-B1; down-regulation of the expression of PCSK9.	[113]
	Palmitic acid (PA)-induced steatosis in HepG2 cells; HFD-induced C57BL/6J mice model	Activating the PI3K/AKT signaling pathway; inhibiting the STING signaling pathway.	[114]
Oxymatrine	High-fat-high-fructose diet-induced Wistar rats model	Improving lipid metabolic disorders, modulating amino acid and bile acid metabolism.	[115]
	PA-induced steatosis in HepG2 cells; HFD/STZ-induced C57BL/6 J mice model	Up-regulation of the expression of SOD, CAT and GSH-Px; down-regulation of the expression of MDA, NLRP3, IL-1β, TGF-β1, and Collagen I.	[116]
	High-fat-high-sucrose diet-induced SD rats model	Activating the PI3K/AKT signaling pathway; up-regulation of the expression of PPARα, ACOX1 and CPT1A.	[117]
Rhynchophylline	OA/PA-induced steatosis in HepG2, AML12 and LMH cells; HFD-induced C57BL/6J mice model	Activating hepatic lipase activity, lipolysis, energy metabolism, and glucose metabolism; inhibiting triglyceride accumulation and oxidative stress.	[118]
Liensinine	PA-induced steatosis in HepG2 cells; HFD-induced C57BL/6 mice model	Activating the TAK1/AMPK signaling pathway; inhibiting the TAK1/NF-κB signaling pathway.	[119]
Neferine	OA-induced steatosis in HepG2 cells, LPS-induced in HepG2 cells and LX-2/hepatic stellate cells; HFD+CCl_4_-induced C57BL/6 mice model	Activating the AMPK/ACC signaling pathway; inhibiting the TLR4/NF-κB p65 and TGFβ-Smad2/3 signaling pathways.	[120]
Sangzhi alkaloids	PA-induced steatosis in HepG2 cells model; HFD-induced C57BL/6 mice model	Activating the AMPK/ACC signaling pathway; up-regulation of the expression of PPARα and PGC1α.	[121]
*p*-Synephrine	HFD-induced C57BL/6J mice model	Activating the AMPK signaling pathway; inhibiting the NF-κB signaling pathway	[122]
Arecoline	HFD-induced SD rats model	Modulating the *Butyricicoccus*/*Christensenella*/*Coriobacteriaceae*-COX2/PGE2 signaling pathway.	[123]
Epiberberine	FFA-induced steatosis HepG2 cells; methionine- and choline-deficient diet-induced C57BL/6J W mice model	Inhibiting the SREBP-1c/FASN signaling pathway; up-regulation of the expression of SHP.	[124]
Tuberostemonine	OA/PA-induced in HepG2 cells; HFD-induced C57BL/6J mice model	Activating mitochondrial function and whole-body energy expenditure; inhibiting lipid deposition and oxidative stress.	[125]
Caffeine	AKT-transfected human hepatoma cells model; insulin-stimulated human hepatoma cells model; AKT transgenic WT FVB/N mice model	Inhibiting the AKT/mTORC1 and SREBP-1/ACC/FASN signaling pathway; inhibition of MyD88 palmitoylation; up-regulation of the expression of NF-κB, IL-6 and TNF-α.	[126]

**Table 5:** Mechanisms of Alkaloids against ALD.

Compound	In Vivo Models	Mechanism of Action	Ref.
Capsaicin	Alcohol-induced acute liver injury C57BL/6 mice model	Activating hepatic antioxidant defense and mitochondrial function; inhibiting matrix metalloproteinase levels.	[127]
	Alcohol-induced liver injury BALB/c mice model	Inhibiting oxidative stress and superoxide generation	[129]
Berberine	Alcohol-induced liver injury ICR mice model	Inhibiting CYP2E1-mediated oxidative stress; enhancing PPARα/PGC-1α-dependent fatty acid β-oxidation; promoting HNF4α/MTTP-mediated lipid export.	[130]
Sinomenine	Alcohol-induced acute liver injury Balb/c mice model	Activating the Nrf2/HO-1 signaling pathway; inhibiting the NLRP3, NF-kB/AKT and MAPK signaling pathways.	[131]

**Table 6:** Mechanisms of Alkaloids against hepatitis.

Compound	In Vitro Models	Mechanism of Action	Ref.
Solanopubamine	HepG2.2.15 cells model; DCFH-induced injury in HepG2 cells	Down-regulation of the expression of HBsAg, HBeAg and Caspase-3/7.	[132]
Acetyl-β-carboline	HepG2.2.15 cells model	Down-regulation of the expression of HBsAg and HBeAg.	[133]
Berberine	HBV-infected HepG2-NTCP cells model	Activating the K^48^-linked polyubiquitination and proteasome-dependent degradation of HNF4α; inhibiting HBV production and cccDNA synthesis.	[134]
	HCVcc cells model; HCVpp cells model; Primary human hepatocytes model	Inhibiting HCV attachment and entry/fusion.	[140]
Sophoridine	HepG2.2.15 cells model	Down-regulation of the expression of HBsAg, HBeAg, p38MAPK, TRAF6, ERK1, NLRP10, and Caspase-1.	[135]
N-methylflindersine	HCVcc cells model; Huh7.5 cells model	Down-regulation of the expression of HCV NS3.	[137]
Pseudane IX	Huh7it-1 cells model; JFH1 cell model	Down-regulation of the expression of HCV NS3.	[138]
Aloperine	Huh7.5 cells model; Primary human hepatocytes model	Inhibiting HCV propagation and entry process; blocking HCV cell-to-cell transmission.	[139]
Neoechinulin B	HCVcc cells model; Primary human hepatocytes model	Inhibiting LXR and LXR-mediated transcription.	[140]

**Table 7:** Mechanisms of Alkaloids against AILD.

Compound	In Vivo Models	Mechanism of Action	Ref.
Koumine	Concanavalin A-induced hepatitis Kunming mice model	Activating the Nrf2/HO-1 signaling pathway; up-regulating the expression of Bcl-2; down-regulating the expression of Keap-1, IL-6, TNF-α, NF-κB, TLR4, TRAF6, and Bax.	[142]
Berberine	BALB/c mouse model	Activating the AMPK signaling pathway; down-regulating the expression of TNF-α, IFN-γ, IL-2, IL-1β, and IL-10.	[143]
Theophylline	Concanavalin A-induced hepatitis White Wistar rats model	Down-regulating the expression of IFN-γ and TGF-β.	[144]
Matrine	Concanavalin A-induced hepatitis ICR mice model	Activating the PI3K/AKT signaling pathway; inhibiting the JNK and JAK1/STAT3 signaling pathways; up-regulation of the expression of SOD, CAT and TAC; down-regulating the expression of TNF-α, NF-κB, IFN-γ, IL-2, IL-6, i-NOS, and COX-2.	[145]

**Table 8:** Clinical translation status of alkaloids discussed in this review.

Compound	Representative Approved Product	Approved or Clinically Used for Liver-Related Diseases	Approved Indication(s)
Berberine/Berberine hydrochloride	Berberine hydrochloride tablets	No	Bacterial enteritis, diarrhea, and other intestinal infectious disorders
Caffeine	Caffeine citrate injection	No	Neonatal apnea; central nervous system and respiratory stimulation in specific clinical settings
Capsaicin	Capsaicin	No	Neuropathic pain, musculoskeletal pain, and topical analgesia
Matrine	Matrine for injection	Yes	Chronic viral hepatitis; adjunctive treatment for liver fibrosis
Oxymatrine	Oxymatrine for injection	Yes	Chronic viral hepatitis; adjunctive treatment for liver fibrosis
Sangzhi alkaloids	Mulberry twig alkaloids tablets	No	Type 2 diabetes mellitus
Sinomenine	Zhengqing fengtongning tablets	No	Rheumatoid arthritis and other inflammatory joint diseases
Theophylline	Theophylline sustained-release tablets	No	Bronchial asthma, chronic obstructive pulmonary disease, and other obstructive airway diseases

## Data Availability

The original contributions presented in this study are included in the article. Further inquiries can be directed to the corresponding authors.

## Abbreviations

The following abbreviations are used in this manuscript:

ABCA1	ATP-binding cassette transporter A1
ABCG1	ATP-binding cassette transporter G1
ACC	Acetyl-CoA carboxylase
ACOX1	Acyl-CoA oxidase 1
ADH	Alcohol dehydrogenase
AIF	Apoptosis-inducing factor
AIH	Autoimmune hepatitis
AILD	Autoimmune liver disease
AKT	Protein kinase B
ALD	Alcoholic liver disease
ALDH	Aldehyde Dehydrogenase
ALT	Alanine aminotransferase
AML12	Alpha mouse liver 12
AMPK	AMP-activated protein kinase
APAP	Acetaminophen
ARE	Antioxidant response element
ASK1	Apoptosis signal-regulating kinase 1
AST	Aspartate aminotransferase
Bak	Bcl-2 antagonist/killer 1
Bax	BCL-2-associated X protein
BBR	Berberine
BSEP	Bile salt export pump
CCL2	C-C motif chemokine ligand 2
cccDNA	Covalently closed circular DNA
CCL4	C-C motif chemokine ligand 4
CD36	Cluster of differentiation 36
CHOP	C/EBP homologous protein
COX-2	Cyclooxygenase-2
Con A	Concanavalin A
CPT1A	Carnitine palmitoyltransferase 1A
CYP1A2	Cytochrome P450 1A2
CYP2D6	Cytochrome P450 2D6
CYP2E1	Cytochrome P450 2E1
CYP3A4	Cytochrome P450 3A4
DAMPs	Damage-associated molecular patterns
DCFH	2′,7′-Dichlorodihydrofluorescein
DILI	Drug-induced liver injury
DNL	De novo lipogenesis
DNA	Deoxyribonucleic acid
EndoG	Endonuclease G
EIF2α	Eukaryotic initiation factor 2 α
ERs	Endoplasmic reticulum stress
ERK1/2	Extracellular signal-regulated kinase 1/2
ETC	Electron transport chain
EZH2	Enhancer of zeste homolog 2
FABP1	Fatty acid-binding protein 1
FASN	Fatty acid synthase
FFA	Free fatty acid
GCLC	Glutamate-cysteine ligase catalytic subunit
GLUT-4	Glucose transporter type 4
GPx	Glutathione peroxidase
GPx4	Glutathione peroxidase 4
GSH	Glutathione
HAV	Hepatitis A virus
HBsAg	Hepatitis B surface antigen
HBV	Hepatitis B virus
HCC	Hepatocellular carcinoma
HCV	Hepatitis C virus
HDV	Hepatitis D virus
HEV	Hepatitis E virus
HFD	High-fat diet
HMGB1	High-mobility group box 1
HNF4α	Hepatocyte nuclear factor 4α
HO-1	Heme oxygenase-1
HSCs	Hepatic stellate cells
HSECs	Hepatic sinusoidal endothelial cells
HSPGs	Heparan sulfate proteoglycans
IFN-γ	Interferon-γ
IL-1β	Interleukin-1β
IL-2	Interleukin-2
IL-6	Interleukin-6
IL-10	Interleukin-10
IRS-1	Insulin receptor substrate 1
JNK	C-Jun N-terminal kinase
LMH	Leghorn male hepatoma
LDLR	Low-density lipoprotein receptor
LPS	Lipopolysaccharide
LXR	Liver X receptor
MASH	Metabolic dysfunction-associated steatohepatitis
MAPK	Mitogen-activated protein kinase
MASLD	Metabolic dysfunction-associated steatotic liver disease
MDA	Malondialdehyde
MEOS	Microsomal ethanol-oxidizing system
mPTP	Mitochondrial permeability transition pore
MyD88	Myeloid differentiation primary response protein 88
NADH/NAD^+^	Ratio of reduced nicotinamide adenine dinucleotide to its oxidized form
NAPQI	N-acetyl-p-benzoquinone imine
NAFLD	Non-alcoholic fatty liver disease
NF-κB	Nuclear factor κB
NLRP3	NOD-like receptor family pyrin domain-containing 3
NLRP10	NOD-like receptor family pyrin domain containing 10
NO	Nitric oxide
Nrf2	Nuclear factor erythroid 2-related factor 2
NS3	Non-structural protein 3
NS5B	Non-structural protein 5B
OA	Oleic acid
ONOO^−^	Peroxynitrite
PA	Palmitic acid
PCD	Programmed cell death
PERK	Protein kinase RNA-like endoplasmic reticulum kinase
PGC-1α	Peroxisome proliferator-activated receptor gamma coactivator 1-α
PI3K	Phosphoinositide 3-kinase
PNPLA3	Patatin-like phospholipase domain-containing 3
PPARα	Peroxisome proliferator-activated receptor α
PPARγ	Peroxisome proliferator-activated receptor gamma
PSC	Primary sclerosing cholangitis
RCR	Rolling circle replication
RNA	Ribonucleic acid
ROS	Reactive oxygen species
SCD1	Stearoyl-CoA desaturase 1
SD	Sprague Dawley
SHP	Small heterodimer partner
SIRT1	Sirtuin 1
SOD	Superoxide dismutase
SREBP	Sterol regulatory element-binding protein
SR-B1	Scavenger receptor class B type 1
STING	Stimulator of interferon genes
SULTs	Sulfotransferases
TAK1	Transforming growth factor-β-activated kinase 1
TCA	Tricarboxylic acid cycle
TGF-β1	Transforming growth factor-β1
TGFβ-Smad2/3	Transforming growth factor-β/Smad2/3 signaling pathway
TH17	T helper 17 cell
TH2	T helper 2 cell
THRSP	Thyroid hormone-responsive spot 14 protein
TLR2	Toll-like receptor 2
TLR4	Toll-like receptor 4
TM6SF2	Transmembrane 6 superfamily member 2
TNF-α	Tumor necrosis factor-α
TORC1	Mechanistic target of rapamycin complex 1
TRAF6	TNF receptor-associated factor 6
TRIM21	Tripartite motif-containing 21
UCP1	Uncoupling protein 1
UGTs	Uridine diphosphate-glucuronosyltransferases
WHO	World Health Organization
